# Oregano essential oil and *Bacillus subtilis* role in enhancing broiler’s growth, stress indicators, intestinal integrity, and gene expression under high stocking density

**DOI:** 10.1038/s41598-024-75533-8

**Published:** 2024-10-25

**Authors:** Ahmed M. Elbaz, Neima K. El-Sonousy, A. Sabry Arafa, M. G. Sallam, Ahmed Ateya, AbdelRahman Y. Abdelhady

**Affiliations:** 1 Nutrition Department, Desert Research Center, Mataria, Cairo, Egypt; 2 Genetics Department, Faculty of Agriculture, Ain Shams University, Cairo, Egypt; 3 Poultry Nutrition Department, Animal Production Research Institute, Agricultural Research Center, Ministry Of Agriculture, Giza, Egypt; 4 Animal Production Department, Agricultural and Biology Research Institute, National Research Centre, Cairo, Egypt; 5 Department of Development of Animal Wealth, Faculty of Veterinary Medicine, Mansoura University, Mansoura, Egypt; 6 Poultry Production Department, Faculty of Agriculture, Ain Shams University, Cairo, Egypt

**Keywords:** Broiler performance, Oregano oil, *Bacillus subtilis*, High stocking density, Gene expression, Immunology, Microbiology, Physiology, Zoology, Diseases

## Abstract

This study investigates the role of dietary *Bacillus subtilis* and oregano essential oil in mitigating the effects of high stocking density on growth performance, carcass traits, physiological stress indicators, gene expression, and intestinal integrity in broiler chickens. A total of, 1250 one-day-old Ross 308 male broiler chicks were randomly allocated to five experimental groups, where each group had five replicates of 50 chicks. Group 1 (control, LSD): 15 chicks/m^2^ fed a basal diet without feed additive, group 2 (HSD): 20 chicks/m^2^ fed a basal diet without feed additive, group 3 (BHSD): 20 chicks/m^2^ fed a basal diet supplemented with *B. subtilis* (500 mg/kg diet), group 4 (OHSD): 20 chicks/m^2^ fed a basal diet supplemented with oregano essential oil (300 mg/kg diet), group 5 (CHSD): 20 chicks/m^2^ fed a basal diet supplemented with oregano essential oil and *B. subtilis*. At 35 days of age, there was a noticeable improvement in the growth performance of broilers fed CHSD under high stocking density through the increase in body weight gain, dressing percentage, and crude protein digestibility with a decrease in feed conversion rate compared to other groups. Adding CHSD enhanced the state of oxidation and immunity through increasing superoxide dismutase, glutathione peroxidase, and the relative weight of bursa of Fabricius, while decreasing malondialdehyde, in addition to increasing plasma triiodothyronine levels. The microbial structure and morphometric parameters improved in the group that received the CHSD compared to the other groups, where villus height and *Lactobacillus* population increased, whereas *Escherichia coli* and *Clostridium perfringens* population decreased. Glucose transporter 2 (GLUT2), fatty acid transporter 1 (FABP1), and amino acid transferase 1 (CAT1) gene expression levels significantly increased when feeding on oregano essential oil with *B. subtilis.* In conclusion, combining oregano essential oil and *B. subtilis* supplements mitigated the effects of high stocking density by enhancing growth performance, antioxidative status, and intestinal integrity, in addition to modifying the genetic expression of genes related to nutrient absorption.

## Introduction

One of the new strategies used to keep pace with the increasing demand for poultry meat is the high stocking density of broiler chickens. In addition to attempting to reduce the poultry industry’s costs, poultry producers and companies have pushed to increase the storage density rate to reduce costs. However, some obstacles affect the spread of this strategy in developing countries, due to the weak technological capabilities of the broiler’s houses, in addition to the presence of some negative effects that include the bird’s exposure to some environmental pressures, slow growth rate, and deterioration of the welfare, immune system, and characteristics of the carcass^1–3^. Many previous studies have reported the possibility of using the high-density strategy under favorable environmental conditions (heat, humidity, and litter) and good husbandry management (ventilation and litter)^4^. In developing countries, it is difficult to provide the optimal environmental conditions to implement the high stocking density strategy due to weak capabilities in addition to environmental pressures such as heat stress (hot weather). This made nutritionists research using some feed additives that reduce the negative effects while the bird is exposed to stress to improve production performance, such as probiotics and plant products^5,6^.

Plant product supplements such as essential oils boost growth performance and feed utilization in the broiler industry^7,8^. Essential oils enhance broiler production by stimulating various digestive enzyme activities, increasing nutrient digestion^9^, reducing the number of intestinal pathogenic microbiota, and improving antioxidant status^10^ and immune response, in addition to preventing subclinical infections^11,12^. The most common essential oils used in broiler diets include turmeric, thyme, cinnamon, garlic, and oregano^13^. Oregano is an herb obtained by drying leaves and flowers of Origanum vulgar. Oregano essential oil has 2 master phenols: thymol and carvacrol, which constitute about 80—85% of essential oil. Using oregano essential oil in the broiler’s diet positively affected growth performance^14^. Supplemental oregano essential oil in the broiler diet exhibited a protective effect against *Clostridium perfringens* by reducing its content in the intestinal^15^.

Probiotics are beneficial bacteria that have positive impacts and that can adjust intestinal bacterial, and the gut environment of the host, as well as, enhance immune response, and resistance to microflora infection, and in turn, improve growth performance in broilers^16,17^. Bacteria *B. subtilis* have been widely used as commercialized probiotic products for feed poultry^18,19^. *B. subtilis* is one type of promising probiotic, due to the high stability of spores, which are resistant to harsh gastrointestinal conditions and high temperatures during feed processing and confer health benefits to the bird^20^. Previous studies indicated that *B. subtilis* had positive impacts on digestibility, intestinal microbes, gut morphology, and immune response^17^ and improved their growth performance^21^. Therefore, the current study hypothesized that using dietary oregano essential oil and *B. subtilis* may improve the performance, carcass traits, gut microflora, physiological stress indicators, and gene expression of broilers subjected to HSD. Therefore, the objective of this study was to evaluate the potential role of adding oregano essential oil and *B. subtilis* on performance, carcass traits, physiological stress indicators, nutrient absorption-related gene expression, and gut microflora and histological in broilers under high stocking density conditions.

## Results

### Productive performance indexes

The impact of supplementation of oregano essential oil with *B. subtilis* and stocking density on productive performance indexes is shown in Table 1. Body weight gain (BWG), the cumulative feed intake (CFI), and feed conversion rate (FCR) during the period from 0 to 21 days were not influenced (*p* < 0.05) by experimental feed additives and stocking density. While the experimental feed additives and stocking density significantly affected BWG and FCR, however, the CFI was not affected during the period from 0 to 35 days. BWG and FCR deteriorated in group HSD compared to the LSD group during the period from 0 to 35 days. Experimental additives improved the BWG and FCR in the BHSD, OHSD, and CHSD groups (*p* < 0.05) compared to the HSD group, during the overall period from 0 to 35 days. Despite this, the best growth performance (BWG and FCR) was in the group fed oregano essential oil with *B. subtilis* (CHSD, *p* < 0.05) compared with BHSD, OHSD, and HSD groups. Nevertheless, BWG and FCR were similar in the chickens that were fed LSD and CHSD. Regarding carcass characteristics, the dressing percentage deteriorated in the HSD chickens group (*p* < 0.05) compared to the LSD chickens group. However, the dressing percentage in the BHSD and CHSD chicken groups was improved than the HSD chicken group. In addition to the decreased abdominal fat content in the BHSD, and CHSD groups (*p* < 0.05) than in the OHSD, HSD, and LSD groups. However, liver weight was not affected in this study.

**Table 1:** Effects of supplementation of oregano essential oil with *B. subtilis* and stocking density on productive performance indexes (growth performance and carcass traits) of broilers.

	LSD	HSD	BHSD	OHSD	CHSD	*P*-value
**Growth performance**						
IBW	41.0 ± 0.12	41.2 ± 0.10	41.3 ± 0.07	41.5 ± 0.14	41.1 ± 0.13	0.820
0-12d						
BWG (g/b)	871 ± 2.54	868 ± 3.65	889 ± 2.71	879 ± 3.56	894 ± 2.84	0.052
CFI (g/b)	1023 ± 5.13	1021 ± 6.12	1022 ± 5.36	1024 ± 4.57	1019 ± 4.62	0.157
FCR (g: g)	1.175 ± 0.02	1.176 ± 0.00	1.149 ± 0.01	1.165 ± 0.00	1.140 ± 0.01	0.076
0-35d						
BWG (g/b)	1965 ± 5.8^a^	1894 ± 4.1^c^	1947 ± 3.5^ab^	1926 ± 4.3^b^	1959 ± 3.6^a^	0.031
CFI (g/b)	3129 ± 7.12	3091 ± 9.15	3121 ± 6.13	3114 ± 8.51	3117 ± 7.62	0.405
FCR (g: g)	1.592 ± 0.02^c^	1.632 ± 0.01^a^	1.603 ± 0.03^bc^	1.617 ± 0.01^b^	1.591 ± 0.02^c^	0.001
SR (%)	96.0	96.0	97.3	96.0	97.3	–
**Carcass traits**						
Dressing (%)	71.5 ± 0.62^a^	69.6 ± 0.94^c^	70.8 ± 1.3^b^	70.1 ± 0.93^c^	71.3 ± 0.72^a^	0.010
Liver	2.74 ± 0.03	2.71 ± 0.01	2.76 ± 0.02	2.69 ± 0.01	2.73 ± 0.06	0.106
Abdominal fat	1.98 ± 0.08^a^	1.96 ± 0.15^a^	1.81 ± 0.06^b^	1.93 ± 0.04^a^	1.76 ± 0.07^b^	0.001

BWG; body weight gain, CFI; cumulative feed intake, FCR; feed conversion rate, LSD; SR; survivability rates, 15 chicks/m^2^ feeding a basal diet without feed additive, HSD; 20 chicks/m^2^ feeding a basal diet without feed additive, BHSD; 20 chicks/m^2^ feeding a basal diet with *B. subtilis*, OHSD; 20 chicks/m^2^ feeding a basal diet with oregano essential oil, CHSD; 20 chicks/m^2^ feeding a basal diet with oregano essential oil and *B. subtilis*, SR (%); survivability rates. Means with different superscripts (a–d) within the same row differ significantly (*p* < 0.05). All data are expressed as the mean ± SD.

## Digestive system performance

The impact of supplementation of oregano essential oil with *B. subtilis* and stocking density on digestive system performance, including nutrient digestibility (%) and digestive enzyme activity are shown in Table 2. Digestibility of nutrients such as dry matter and fat was not influenced (*p* < 0.05) by experimental additives or stocking density, except for crude protein, which increased in the CHSD group compared to other groups. Trypsin enzyme activity increased in CHSD and OHSD groups (*p* < 0.05) compared to LSD, HSD, and BHSD groups. However, lipase and amylase enzyme activity was not affected (*p* < 0.05) by the experimental feed additions and stocking density.

**Table 2:** Effects of supplementation of oregano essential oil with *B. subtilis* and stocking density on digestive system performance (nutrient digestibility (%) and digestive enzymatic activity (U/ml)) of broilers.

	LSD	HSD	BHSD	OHSD	CHSD	*P*-value
**Nutrient digestibility**						
Dry matter	76.8 ± 1.07	76.4 ± 0.85	77.2 ± 1.62	76.6 ± 0.74	77.1 ± 0.91	0.187
Crude protein	83.5 ± 0.86^b^	81.7 ± 1.52^c^	82.9 ± 0.75^b^	83.2 ± 0.91^b^	84.8 ± 0.63^a^	0.001
Ether extract	64.1 ± 0.09	63.9 ± 0.06	64.3 ± 0.11	64.2 ± 0.07	64.7 ± 0.05	0.194
**Digestive enzymatic**						
Trypsin	23.2 ± 0.03^b^	22.8 ± 0.02^b^	23.1 ± 0.05^b^	23.8 ± 0.01^a^	24.1 ± 0.02^a^	0.001
Amylase	5.17 ± 0.03	5.09 ± 0.08	5.13 ± 0.09	5.11 ± 0.02	5.16 ± 0.05	0.112
Lipase	7.35 ± 0.10	7.32 ± 0.04	7.58 ± 0.14	7.29 ± 0.08	7.61 ± 0.12	0.056

LSD; 15 chicks/m^2^ feeding a basal diet without feed additive, HSD; 20 chicks/m^2^ feeding a basal diet without feed additive, BHSD; 20 chicks/m^2^ feeding a basal diet with *B. subtilis*, OHSD; 20 chicks/m^2^ feeding a basal diet with oregano essential oil, CHSD; 20 chicks/m2 feeding a basal diet with oregano essential oil and *B. subtilis*. Means with different superscripts (a–c) within the same row differ significantly (*p* < 0.05). All data are expressed as the mean ± SD.

## Physiological stress indicators

The impact of supplementation of oregano essential oil with *B. subtilis* and stocking density on physiological stress indicators, including antioxidative status and triiodothyronine (T3), are shown in Table 3. SOD levels increased (*p* < 0.05) in CHSD and BHSD groups compared with other groups. Furthermore, GPx levels increased in LSD and CHSD groups compared with other groups, while MDA levels decreased in OHSD and CHSD groups compared with other groups. Plasma triiodothyronine (T3) levels also increased in the BHSD and CHSD (*p* < 0.05) groups compared with other groups. The immune organs were not affected, including the spleen and thymus, by the experimental additives and the stocking density (Table 4), except for the relative weight of the bursa of Fabricius, which increased in in BHSD and CHSD groups (*p* < 0.05) compared with other groups.

**Table 3:** Effects of supplementation of oregano essential oil with *B. subtilis* and stocking density on physiological stress indicators (antioxidative status and triiodothyronine (T3)) and lymphoid organs (%) of broilers.

	LSD	HSD	BHSD	OHSD	CHSD	*P*-value
**Physiological stress indicators**						
MDA (nmol/L)	4.28 ± 0.05^b^	4.74 ± 0.02^a^	3.21 ± 0.03^b^	4.03 ± 0.01^c^	3.36 ± 0.04^c^	0.001
SOD (U/mL)	7.11 ± 0.04^b^	6.45 ± 0.08^d^	7.06 ± 0.06^b^	6.87 ± 0.11^c^	7.35 ± 0.05^a^	0.024
GPx (U/mL)	142 ± 0.32^a^	127 ± 0.19^c^	138 ± 0.43^b^	131 ± 0.35^c^	144 ± 0.21^a^	< 0.001
T3 (ng/ml)	1.19 ± 0.17^b^	1.14 ± 0.23^b^	1.43 ± 0.34^a^	1.22 ± 0.19^b^	1.51 ± 0.16^a^	0.001
**Lymphoid organs**						
Spleen	0.22 ± 0.01	0.21 ± 0.01	0.23 ± 0.03	0.22 ± 0.01	0.23 ± 0.02	0.215
Thymus	0.34 ± 0.04	0.32 ± 0.12	0.35 ± 0.06	0.33 ± 0.03	0.36 ± 0.04	0.074
Bursa of Fabricius	0.15 ± 0.00^ab^	0.11 ± 0.01^b^	0.19 ± 0.00^a^	0.14 ± 0.00^ab^	0.21 ± 0.01^a^	0.030

MDA; malondialdehyde, SOD; superoxide dismutase, GPx; glutathione peroxidase, T3; triiodothyronine, LSD; 15 chicks/m^2^ feeding a basal diet without feed additive, HSD; 20 chicks/m^2^ feeding a basal diet without feed additive, BHSD; 20 chicks/m^2^ feeding a basal diet with *B. subtilis*, OHSD; 20 chicks/m^2^ feeding a basal diet with oregano essential oil, CHSD; 20 chicks/m^2^ feeding a basal diet with oregano essential oil and *B. subtilis*. Means with different superscripts (a–c) within the same row differ significantly (*p* < 0.05). All data are expressed as the mean ± SD.

**Table 4:** Effects of supplementation of oregano essential oil with *B. subtilis* and stocking density on intestinal integrity (microbial architecture and histomorphology) of broilers.

	LSD	HSD	BHSD	OHSD	CHSD	*P*-value
**Microbial architecture**						
E. coli	3.17 ± 0.36^c^	4.52 ± 0.45^a^	3.14 ± 0.29^c^	4.08 ± 0.32^b^	2.96 ± 0.26^c^	< 0.001
Salmonella	1.06 ± 0.00	1.16 ± 0.01	0.98 ± 0.00	1.22 ± 0.00	1.01 ± 0.02	0.062
C. perfringens	6.51 ± 0.12^b^	7.32 ± 0.07^a^	6.47 ± 0.09^b^	7.15 ± 0.08^a^	6.38 ± 0.06^b^	0.039
Lactobacillus	5.24 ± 0.03^b^	4.63 ± 0.01^d^	5.31 ± 0.01^b^	4.96 ± 0.02^c^	5.94 ± 0.01^a^	0.001
**Histomorphology**						
Villus height (µm)	562 ± 1.62^c^	534 ± 2.01^c^	827 ± 1.80^a^	691 ± 2.15^b^	908 ± 2.43^a^	< 0.001
Crypt depth (µm)	78.4 ± 0.87	78.2 ± 1.32	80.4 ± 0.95	80.7 ± 0.68	81.3 ± 1.11	0.068
VH/CD ratio	7.12 ± 0.76^d^	6.85 ± 0.61^d^	10.2 ± 0.45^b^	8.56 ± 0.70^c^	11.1 ± 0.52^a^	< 0.001

LSD; 15 chicks/m^2^ feeding a basal diet without feed additive, HSD; 20 chicks/m^2^ feeding a basal diet without feed additive, BHSD; 20 chicks/m^2^ feeding a basal diet with B. subtilis, OHSD; 20 chicks/m^2^ feeding a basal diet with oregano essential oil, CHSD; 20 chicks/m^2^ feeding a basal diet with oregano essential oil and *B. subtilis*. *E. coli*; *Escherichia coli*, *C. perfringens*; *Clostridium perfringens*. Means with different superscripts (a–c) within the same row differ significantly (*p* < 0.05). All data are expressed as the mean ± SD.

## Intestinal integrity

The impact of supplementation of oregano essential oil with *B. subtilis* and stocking density on intestinal integrity, including microbial architecture and histomorphology, are shown in Table 4. The experimental additions led to significant changes in the microbial content through a noticeable decrease in the intestinal content of *E. coli* and *C. perfringens* in the CHSD and BHSD groups (*p* < 0.05) compared to the OHSD and HSD groups, whereas, the *Lactobacillus* count increased in the CHSD group compared with other groups. However, *Salmonella* counts were not influenced (*p* < 0.05) by the experimental additions or stocking density. Villus height increased in the CHSD and BHSD groups (*p* < 0.05) compared to the other groups. Furthermore, the VH/CD ratio was higher in the CHSD group than in the rest groups, however, the crypt depth was not influenced between the experimental treatments.

## Gene expression

**Fig. 1:**
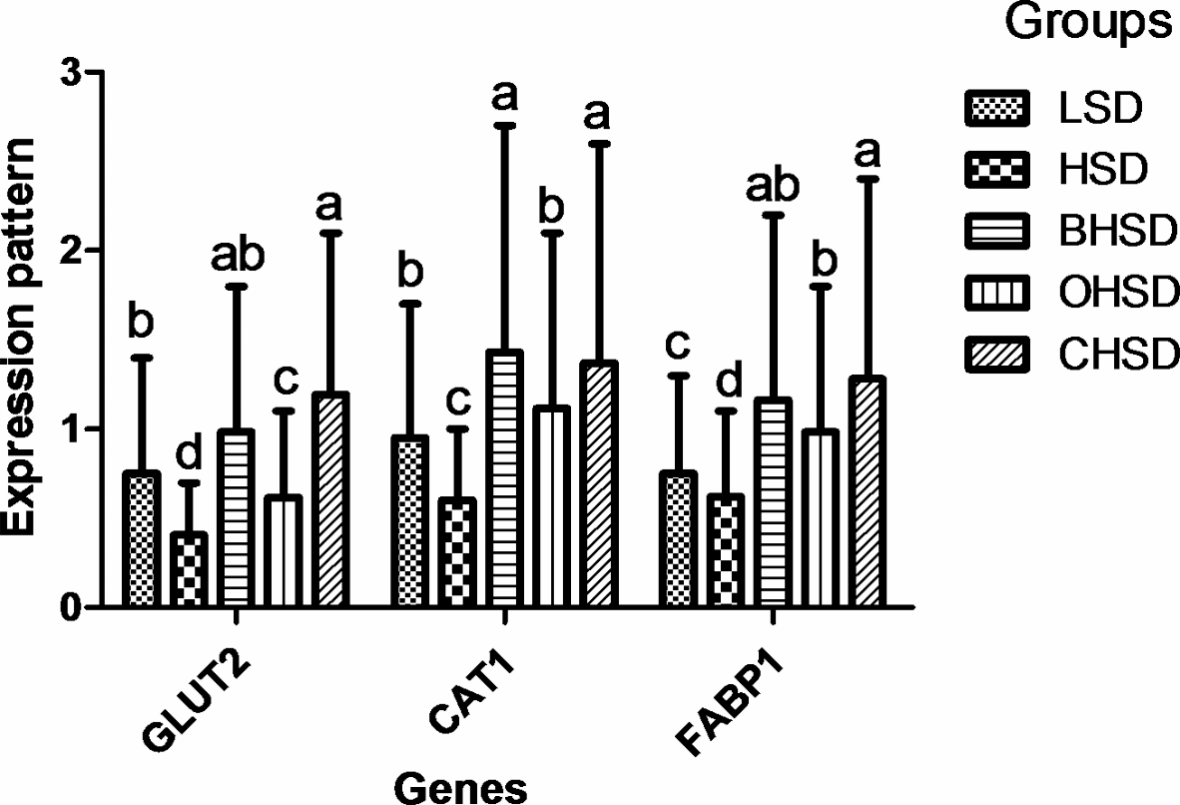
Effect of adding oregano essential oil and *Bacillus subtilis* on glucose transporter 2 (GLUT2), amino acid transferase 1 (CAT1), and fatty acid transporter 1 (FABP1) gene expression of broiler chickens under high stocking density. LSD; 15 chicks/m^2^ feeding a basal diet without feed additive, HSD; 20 chicks/m^2^ feeding a basal diet without feed additive, BHSD; 20 chicks/m^2^ feeding a basal diet with *B. subtilis*, OHSD; 20 chicks/m^2^ feeding a basal diet with oregano essential oil, CHSD; 20 chicks/m^2^ feeding a basal diet with oregano essential oil and *B. subtilis*. Data are presented as the mean values with their standard errors. Values with different superscript letters are significantly different (*P* < 0.05). All data are expressed as the mean ± SD. *Beta*-actin was used as a housekeeping gene.

The impact of supplementation of oregano essential oil with *B. subtilis* and stocking density on gene expression, including GLUT2, FABP1, and CAT1, are shown in Fig. 1. In the HSD group, GLUT2, CAT1, and FABP1 gene expression significantly down-regulated (*p* < 0.05) than that in the LSD group. However, in the BHSD and CHSD groups, GLUT2, CAT1, and FABP1 gene expression significantly up-regulated (*p* < 0.05) than in the HSD group. Furthermore, the OHSD group had higher expression of FABP1 gene (*p* < 0.05) than the LSD group.

## Discussion

Poultry industry suffers from many stress factors that harm production performance and costs, including rearing stress, such as high stocking density, which producers frame to reduce costs and provide the required protein needs. Therefore, this study investigated the potential positive effect of nutritional additives to mitigate the harmful effects of high stocking density on growth performance, carcass traits, indicators of physiological stress, intestinal integrity, and some gene expressions related to nutrient absorption of broilers.

Results of the current study display an impairment in growth performance with an increasing stocking density through a decrease in body weight and an increase in the feed conversion ratio. Consistent with our results, several studies have detected a significantly lower growth performance with increasing stocking density^22,23^. Similarly, Nasr et al.^24^ reported increasing stocking density had a decrease in body weight, resulting in a detrimental impact on growth performance, which supported the current results. In addition, our results display a noticeable amelioration in growth performance in the broiler fed combination of oregano essential oil and *B. subtilis* under conditions of high stocking density. Our results are supported by many studies, which reported that adding *B. subtilis* to broiler diets led to a noticeable improvement in growth performance^19,25^. Our findings are consistent with past studies that have shown that feeding oregano essential oils to broilers under environmental stress improves growth performance^26,27^. Likewise, Basmacioğlu et al.,^28^ and Zaazaa et al.,^14^ reported that adding oregano essential oil improved the performance of broiler chickens by higher body weight gain and the lowest feed conversion ratio. The noticeable improvement in the productive performance of chickens fed oregano essential oil and *B. subtilis* combination under high stocking density conditions may be due to promoting nutrient utilization, enhancing digestive and antioxidative enzymes, and immune response, in addition to reducing the number of pathogenic microorganisms and improving intestine morphology, as indicated by Jang et al.^29^; Alagawany et al.^30^; Elbaz et al.^9^. The results of our study are consistent with previous reports, which demonstrate the positive effect of adding oregano essential oil and *B. subtilis* on improving growth performance. Therefore, combining oregano essential oil with *B. subtilis* synergized in improving growth performance.

In agreement with the results of growth performance in the current study, there was an improvement in carcass specifications through an increase in the dressing percentage in the group fed a mixture of oregano essential oil with *B. subtilis* compared to the group that did not receive additives under high stocking density. However, there was a noticeable similarity in carcass characteristics between the group fed a mixture of oregano essential oil with *B. subtilis* and the group with low stocking density. Many studies have confirmed a close connection between enhanced nutrient digestibility and increased dressing percentage in broilers via significant increases in gut percentage and length of the intestine, in addition to modifying the microbial content^31,32^. Results by Jang et al.^29^ and Elbaz et al.^9^ reported that phytogenic (including essential oil) or probiotic feed additives improved the apparent intestinal digestibility of nutrients and promoted digestive enzyme activity, reflecting on increased dressing percentage.

Moreover, in this study, abdominal fat content decreased in chickens fed *B. subtilis*, whether alone or combined with oregano essential oil. These results agree with Elbaz et al.^33^, who reported adding probiotics to broiler chicken diets to reduce abdominal fat percentage relative to BW. Likewise, feeding broilers a diet containing essential oil led to decreased abdominal fat^34^. The decrease in abdominal fat could be related to the active chemicals in essential oils that are involved in lipid metabolism resulting in a reduced rate-limiting enzyme involved in cholesterol synthesis, 3 hydroxy-3‐methylglutaryl coenzyme A reductase, which affects cholesterol production and reduces abdominal fat^35^. Beneficial microbes (including *B. subtilis*) in the intestine play an important role in the metabolism of fats by reducing the production of the acetyl-CoA carboxylase enzyme, which has a catalytic impact in the synthesis of fatty acids, which lessens lipogenesis^9^. The decrease in abdominal fat has an economic benefit for the poultry industry, as the decrease in abdominal fat corresponds to an increase in the percentage of carcass yield, in addition to the fact that abdominal fat is one of the main wastes in slaughterhouses^36^. Abdominal fat lower suggests the beneficial influence of the experimental supplement on the distribution of fat in the carcass, which may contribute to the fatty acids between the muscles and thus improve carcass quality^37^. Subsequently, a combination of *B. subtilis* and oregano essential oil enhances the characteristics of the carcass, which includes increasing the dressing percentage and improving the distribution of the fat inside the carcass, which enhances the carcass quality.

Digestive system performance was investigated to clarify the effect of experimental additives, via nutrient utilization, and enzyme activity was evaluated. The current research showed a noticeable improvement in the digestibility of crude protein and increased endogenous excretion of trypsin enzyme in chickens fed CHSD, in line with findings from previous studies^24,38^. These results are consistent with some reports that feeding broiler chickens a diet containing probiotics enhanced nutrient digestion^5^. Some results of previous studies also showed that *B. subtilis* increased the metabolism rates of crude protein and utilization rate^19^. A study by Basmacioğlu et al.^28^ confirmed that adding oregano essential oil improved the digestibility of crude protein and increased chymotrypsin activity. In agreement with the findings, Lee et al.^39^ noted that thymol and cinnamaldehyde compounds increased digestive enzyme activities in broilers’ pancreas tissue and intestinal digesta at 21 d. Our results demonstrate the beneficial effect of adding a mixture of oregano essential oil and *B. subtilis* on enhancing nutrient digestion and secretion of endogenous enzymes in broilers, which mitigates the harms of high storage density.

In this study, experimental additives enhanced oxidative status and thyroid activity, in addition to boosting the immune response by increasing SOD, T3 levels, and the relative weight of the bursa of Fabricius and decreasing MDA levels compared to the HSD group. These results are consistent with some reports that essential oil or probiotic positively impact antioxidant status, including catalase, GPx, and SOD and prevent the formation of reactive oxygen species and fatty acid oxidation^40–42^. In other studies^43,44^, in which essential oil was added, including cinnamon, carvacrol, and thymol, enhancement in antioxidant activity was noted by decreased MDA levels and increased total SOD activity in the liver. Zhang et al.^45^ noted that adding *B. subtilis* enhanced the antioxidant capacity of broilers. Similarly, Abdel-Moneim et al.^46^ demonstrated that probiotics substituted for antibiotics resulted in increased serum triiodothyronine (T3) concentrations and enhanced immunity in broilers. Thyroid hormones play an important role in stimulating the synthesis of some enzymes, hormones, and structural proteins, and elevated thyroid hormone levels following probiotic administration enhance chicken metabolism *48*. Adding a mixture of essential oil with *B. subtilis* to a broiler diet positively impacts growth performance through antioxidant properties and immunomodulatory effects.

The gut contributes to maintaining the bird’s performance and immunity through the digestion and absorption of nutrients, in addition to its role in immune functions via maintaining the gut barrier functions, the condition of the villus, and the microbial content. The villus height and depth crypt are the most critical measures of the intestine’s absorption ability^47–49^. Therefore, studying the intestinal microbial content and morphology was necessary to clarify the effect of experimental additives on intestinal health. In the current study, feeding on a mixture of oregano essential oil and *B. subtilis* resulted in enhancing the integrity of the intestine, as the villi length and the number of lactobacilli increased, in contrast, the number of *E. coli* and *C. perfringens* decreased. These results are consistent with many reports that adding essential oil or probiotics positively impacts gut status via an increase in beneficial microbes and a decrease in harmful microbes^16,41^. Similarly, numerous scientists have stated that adding essential oils to the diet can increase the length and depth of the villi in the small intestine, resulting in improved absorption of nutrients^50,51^. The enhanced histomorphological appearance of the small intestine’s mucosa, characterized by heightened villus height, could be attributed to the bioactive substances found in essential oils that protect villi from damage by enhancing antioxidant enzyme function^52^. In addition, some research indicated that *B. subtilis* has bacteriostatic action on common pathogens like as *E. coli* due to *B. subtilis* secretion of pathogen-suppressive some substances^53^. *B. subtilis* are aerobes that need large amounts of oxygen for growth and reproduction, which could suppress the growth of aerobes like *E. coli*, thus maintaining the balance of the intestinal microenvironments^19^. *B. subtilis* and oregano essential oil can maintain the balance of the gut microbes by maintaining intestinal beneficial bacteria, in addition to competing with pathogens for nutrients with increased absorption surface, thus enhancing the feed conversion ratio and growth.

To evaluate environmental stress impacts different parameters have been used, including blood metabolites, and changes in microbial content, in addition, recent developments have focused on analyzing gene expression to identify genes important in stress responses. Different defensive measures are stimulated to protect tissue cells from stress, such as activating stress response genes, thus, gene regulation during stress is a potential indicator of stress severity. Exposure of the bird to stress impairs the absorption of nutrients by modulating the gene expression responsible for nutrient transport, for example, genes of the *GLUT2*, *FABP1*, and *CAT1*. *GLUT2* gene, which is responsible for the transfer of fructose and glucose into portal blood capillaries in broiler intestines *55*. In addition, decreased intestinal expression of the *FABP1* in stressed broilers, which is involved in fatty acid uptake and transport, was observed^55^. The *FABP1* gene’s function is to uptake long-chain fatty acids into enterocytes, which are primarily expressed in the intestinal epithelium. Additionally, stressed birds were found to change the expression of the *CAT1*^56^. Our results showed a decrease in *FABP1* and *CAT1* gene expression in the HSD group, as a result of the bird being exposed to high stocking density (environmental stress). The current study’s findings align with previous research by Habashy et al.^55^ which showed that heat stress lowers *FABP*1 expression in the gut of broilers. Furthermore, the expression of the *FABP1*, *GLUT2*, and *CAT1* genes increased in the broiler fed a diet that included oregano essential oil with B. subtilis under HSD compared with other groups. Adding probiotic strains to rabbit’s diet has been previously shown to enhance nutrient absorption^57^. Additionally, the expression of protein and glucose transporters genes showed upregulation in broilers feeding on probiotics^58^. Some study data indicated that FABP-2, SGLT-1, GLUT2, and CAT-1 gene expression levels were significantly increased (*p* < 0.05) in rabbits on a diet included with probiotics^54^. Thus, adding a mixture of oregano essential oil and *B. subtilis* regulates the gene expression of cells specialized in nutrient absorption, which enhances the utilization of nutrients and results in improved growth performance under high stocking density.

## Conclusion

Our results demonstrate the harmful effect of high stocking density on productive performance, immune and antioxidative status, and intestinal integrity, in addition, the gene expression of nutrition absorption-related genes in the intestine decreased. The synergistic influence of the mixture of oregano essential oil and *B. subtilis* stimulates the digestion and absorption of nutrients; in addition, it enhances the immune response and oxidative status, thus improving growth performance in broilers under high stocking density. Oregano essential oil with *B. subtilis* mixture enhanced intestinal integrity by increasing beneficial microbes and villus length, in addition to modifying the genetic expression of genes related to nutrient absorption. Therefore, we recommend using it as a nutritional additive to mitigate the effects of high stocking density stress.

## Materials and methods

### Experimental design and diets

In the current study, 1250 one-day-old male broiler chicks (ROSS 308) were randomly divided into five experimental groups of 250 chicks each, and each group contained 5 replicates. The experimental groups were as follows; group 1 (control, LSD): 15 birds/m2 feeding a basal diet without feed additive, group 2 (HSD): 20 birds/m2 feeding a basal diet without feed additive, group 3 (BHSD): 20 birds/m2 feeding a basal diet supplemented with *B. subtilis* (500 mg/kg diet), group 4 (OHSD): 20 birds/m2 feeding a basal diet supplemented with oregano essential oil (300 mg/kg diet), group 5 (CHSD): 20 birds/m2 feeding a basal diet supplemented with oregano essential oil with *B. subtilis*. The diets were formulated to meet the needs of chicks as recommended in NRC^59^ and the diets were mixed in two batches (the starters and the finishers, as given in Table 5) and stored in bags at room temperature till starting the experiment. The broiler house temperature was closely monitored, starting at 32.5 C on two days and decreasing by 1 C every 3 days till 22 C end of the experiment. The chicks were exposed to 24 h of lighting for the first 1 week and then 2 h of darkness and 22 h of lighting until the end of the experiment, with an average light intensity of 20 lx. Chicks had access to water and feed around the clock. Oregano essential oil was provided by ELHAWAG Company (Giza, Egypt). *B. subtilis* strains (1.5 × 10^5^ CFU/g feed) were obtained from the Department of Microbiology, Faculty of Agriculture, Ain Shams University, Egypt.

**Table 5:** Ingredients and nutrient composition of experimental diets.

Item	Starter	Finisher
Corn	55.35	58.00
Soybean meal (48%)	32.50	27.50
Sunflower meal	3.00	3.30
Corn gluten meal	3.00	3.00
Soybean oil	2.00	4.00
Calcium carbonate	1.40	1.50
Dicalcium phosphate	2.00	1.90
DL-Methionine	0.15	0.20
Salt	0.25	0.25
Premix*	0.25	0.25
Sodium bicarbonate	0.10	0.10
Calculated composition		
ME (kcal/kg)	3000	3150
Crude protein (%)	23	21
Calcium (%)	1.071	1.068
Available P (%)	0.493	0.471

* Premix provides per kg of diet: vitamin K, 0.004 g; vitamin A, 14,000 IU; vitamin E, 0.05 g; vitamin D3, 3000 IU; pyridoxine, 0.003 g; pantothenic acid, 0.02 g; cobalamin, 0.006 g; choline, 0.15 g; niacin, 0.06 g; folic acid, 0.0002 g; Ca, 0.048 g; P, 0.00032 g; Mn, 0.1 g; Zn, 0.08 g; Fe, 0.05 g; Cu, 0.01 g; iodine, 0.000015 g; Co, 0.000025 g.

### Productive performance indexes

The initial average body weight was 41.2 ± 3 g and the average body weight of broiler chicks from each group was recorded. The average weight and feed intake were recorded weekly. The performance indexes were evaluated by calculating the body weight gain (BWG), the cumulative feed intake (CFI), the feed conversion rate (FCR, as CFI (g) per mean BW (g) for each replicate of the experimental groups), and survivability rates (%). Carcass traits were calculated based on relative live body weight, including dressing percentage, abdominal fat, and liver, in addition to an index of the chick’s immune situation including the spleen, thymus, and bursa of Fabricius.

### Digestive system performance

The performance of the digestive system including nutrient digestibility, and digestive enzymatic activity was evaluated, by ten broilers from each group were collected at the age of 35 days and placed individually in digestion cages to begin the digestion experiment. Excreta were collected every eight hours for 3 days. At the end of the experiment, the excreta was dried and stored, then the feed was collected to measure nutrient digestibility (crude protein (CP), dry matter (DM]), and ether extract (EE)) according to AOAC^60^. During slaughter at 35 days, samples of cecal contents of about 2 g (10 birds/ group) were collected and placed in a neutral saline solution for preservation till analysis. The supernatant part after the solution was separated through centrifugation (1792 g for 15 min) to estimate digestive enzyme activity, including trypsin^61^, amylase^62^, and lipase^63^.

### Physiological stress indicators

Before slaughter (35 days), ten blood samples were taken from each group from the jugular vein in heparinized tubes to obtain plasma and then centrifuged (3000×g for 15 min). Oxidation status indications in plasma were examined, including glutathione peroxidase (GPx), superoxide dismutase (SOD), and malondialdehyde (MDA), by using commercial kits (Spinreact Co. Girona, Spain). Plasma triiodothyronine (T3) hormone level was estimated by radioimmunoassay with a kit produced by the Institute of Isotopes Co., Ltd. (Budapest, Hungary).

### Intestinal integrity

Intestinal integrity including intestinal microbial architecture and histomorphological were evaluated. During slaughter, about 2 g of caecal contents were collected to determine the intestinal microbiota diversity. Samples were put into sterilized tubes and diluted with sterilized water to 1:10, then plated on agar plates using a glass rod, and then incubated for 48 h. Eosin methylene blue agar medium, Salmonella-Shigella agar medium, Shahidi Ferguson Perfringens (SFP) agar, and MacConkey agar medium were used for culturing Escherichia coli (37◦C), Salmonella (37◦C), Clostridium perfringens (37◦C), and Lactobacillus (30◦C), respectively. Results microbial count were evaluated as log10 colony-forming units per gram of cecal digesta. Ileal samples (~ 3 cm) were collected during slaughter to measure histomorphological. The samples were placed in 10% formalin saline solution till analyzed. Ileal slides (3–4 μm thickness) were cut using a rotary microtome, then checked by optical microscope to detect the histomorphological changes (villus height (VH) and crypt depth (CD)), as described by Elbaz et al. *5*.

### Gene expression

Some genes associated with nutrient absorption were selected, including glucose transporter 2 (GLUT2), fatty acid transporter 1 (FABP1), and amino acid transferase I (CAT1) with beta-actin (β-actin). According to the manufacturer’s instructions, total RNA was isolated from the ileum mucosa (two birds/ replicates) using the Trans-Zol reagent (Beijing, China). Using the Revert Aid First Strand cDNA Synthesis kit (Thermo Fisher Scientific, Vilnius, Lithuania) the cDNA was synthesized from total RNA, as directed by the manufacturer’s instructions. Primer sequences for the genes selected for the current study were synthesized by Invitrogen (ThermoFisher Scientific, Vilnius, Lithuania). The forward and reverse primers for FABP1 were ACTGGCTCCAAAGTAATGACCAATG and TGTCTCCGTTGAGTTCGGTCAC (Accession number NM_204192.4); for CAT1: CATCAACATCCTCGTCATC and CTCCATCCCAACCTACATAC (Accession number EU360441.1); for GLUT2: AGAGGAAACTGTGACCCGATGA and AACGAAGAGGAAGATGGCGA (Accession number Z22932.1); for βeta-actin: CCACCGCAAATGCTTCTAAAC and AAGACTGCTGCTGACACCTTC (Accession number NM 205518.2), respectively. After the forty cycles, a melting-curve analysis confirmed that a gene product was amplified. After the amplification procedure, the PCRs were carried out in a real-time thermal cycler machine (Rotor-Gene Q, QIAGEN, Hilden, Germany). Amplification curves and the RT-PCR data were analyzed using Rotor-Gene Q Series Software automatically PCR data obtained. In addition, the relative gene expression in samples was determined by comparing the CT value of each sample to that of the positive control according to Yuan et al.^64^.

### Statistical analysis

The effects of the experimental supplemental on the performance, physiological stress indicators, microbial architecture, histomorphology, and gene expression were assessed using an ANOVA as a completely randomized design (GLM procedure in SAS Statistical Analysis Software, version 9.1^65^), . Differences among means in the presence of statistical differences were assessed (*p* < 0.05) using Duncan’s multiple-range test. All data are expressed as the mean ± SD.

## Data Availability

All data generated or analyzed during this study are included in this published article.

## Abbreviations

LSD: 15 birds/m^2^ feeding a basal diet without feed additive
HSD: 20 birds/m^2^ feeding a basal diet without feed additive
BHSD: 20 birds/m^2^ feeding a basal diet supplemented with B. subtilis
OHSD: 20 birds/m^2^ feeding a basal diet supplemented with oregano essential oil
CHSD: 20 birds/m^2^ feeding a basal diet supplemented with oregano essential oil with B. subtilis
GPx: Glutathione peroxidase
SOD: Superoxide dismutase
MDA: Malondialdehyde
T3: Triiodothyronine
GLUT2: Glucose transporter 2
FABP1: Fatty acid transporter 1
CAT1: Amino acid transferase 1
CP: Crude protein
DM: Dry matter
EE: Ether extract
BWG: Body weight gain
CFI: Cumulative feed intake
FCR: Feed conversion rate
B. subtilis: Bacillus subtilis
